# Revision surgery for osteochondral lesions of the talus: Are there predictive factors for successful surgery?

**DOI:** 10.1186/s13018-026-07264-7

**Published:** 2026-10-27

**Authors:** Christoph Biehl, Thaqif El Khassawna, Carina Fritzsche, Nike Walter, Lotta Biehl, Christian Heiss, Markus Rupp

**Affiliations:** 1 Experimental Trauma Surgery, Justus-Liebig-University of Giessen, Giessen, Germany; 2 Department of Trauma, Hand and Reconstructive Surgery, University Hospital Giessen GmbH, Rudolf-Buchheim-Str. 7, 35392 Giessen, Hesse Germany; 3 Clinic and Polyclinic of Surgery, University Hospital Regensburg Clinic and Polyclinic for Surgery, Regensburg, Germany; 4 Medical Faculty, Heidelberg of Heidelberg University, Heidelberg, Germany; 5 School of Pharmacy, The University of Jordan, Amman, 11942 Jordan; 6 Adjunct Professor for Orthopedics, Biruni University, Istanbul, Turkey

**Keywords:** Ankle joint revision, Osteochondral lesion, Foot and ankle, Postoperative rehabilitation time, Pain management

## Abstract

**Purpose:**

Osteochondral lesions of the talus (OLT) are common injuries of the ankle associated with persistent pain and functional impairment. Revision procedures are frequent. Currently, objective criteria to guide revision decision-making remain limited. This retrospective study is designed to identify factors associated with patient-reported outcome after surgery pathways and to enhance selection of patients who may benefit from revision surgery in OLT.

**Methods:**

Between 2015 and 2018, 64 patients underwent surgical treatment for OLT (97 procedures in total). A symptomatic subgroup of 21 patients required additional procedures (33 revision operations). Outcomes at a mean follow-up of 3 years were assessed using the Visual Analog Scale (VAS), the American Orthopaedic Foot and Ankle Society (AOFAS) score, and the Foot and Ankle Outcome Score (FAOS). Primary outcome was a patient-level PROM-based classification (Success/ Partial relief/No relief) at follow-up. Analyses were performed per patient; procedure counts were reported descriptively.

**Results:**

Of 64 patients, 43 (67%) achieved a successful outcome after a single procedure, whereas 21 (33%) required multiple procedures. In the multiple-surgery group, 9/21 (43%) were classified as Success at follow-up. PROM categories in follow up correlated significantly with AOFAS, VAS, and FAOS. A longer interval (> 24 months) between procedures might be associated with a higher likelihood of a *habituation effect* at follow-up.

**Conclusion:**

PROM-based classification provides a patient-centered assessment of outcome after revision pathways for OLT. The FAOS score subdomains proved particularly valuable in identifying specific issues that could justify a revision. The waiting time between surgeries appears relevant and should be considered when planning revision surgery. Our data suggests that a longer interval between surgeries (exceeding 24 months) might be associated with habituation effects and adaptation processes, leading to a reduction in periarticular swelling and the continuation of physiotherapy. Despite moderate success rates, the treatment of OLT remains challenging, and preoperative factors such as age and activity level should be considered when evaluating the chances of success.

## Introduction

Osteochondral lesions (OCL) of the talus (OLT) often occur because of ankle injuries. The latter account for around 15% of all sports injuries [1]. This makes the ankle joint the third most affected joint after the knee and elbow joints [2]. Up to 6% of ankle sprains are associated with osteochondral fractures, particularly of the talus [3–5].

Microtrauma, congenital factors, and metabolic disorders have also been described as causes of OCL. Persistent pain and functional impairment affect quality of life, which is why stage-appropriate treatment is recommended [6]. To date, no single treatment method has been shown to be clearly superior [7, 8]. The challenges in treating OCL are compounded by the size, location, and stage of the lesion, as well as patient-specific factors such as age and activity level [9, 10]. Conservative therapy is usually used in the early stages of the injury [11]. In literature, evaluated treatment concepts for “return to work or sports” after operation in OCL of the talus are difficult to find, and rehabilitation protocols are the subject of current scientific debate [12–14].

Patients often report persistent pain and limitations after surgical treatment. These complaints are usually non-specific and improve over time, meaning repeat surgery is not necessary. However, in some patients, the complaints persist permanently. Revision surgery cannot be justified based on complaints alone. Currently, there are no clear, objective criteria to suggest that further surgery would benefit the patient. With the exception of graft failure detectable by MRI in a symptomatic patient. The decision to perform revision surgery is often based on subjective information provided by the patient, but this should be supported by objective findings. Imaging is not a definitive criterion within the first year after surgery, as remodeling takes time. Many patients show similar MRI scan results without complaining of symptoms. Revision surgery is usually considered if the pain is projected onto the OLT and the MRI scan shows progression of the condition. Most surgeons then opt for a combination of cellular (cancellous bone) and acellular (autologous matrix-induced chondrogenesis, AMIC) materials. In addition, capsular fibrosis and scarring can lead to pain and functional limitations in joint movement. There is not always a connection with the OCL of the talus, and a change in procedure at the former lesion is usually unnecessary. Scores such as the American Orthopedic Foot and Ankle Society (AOFAS) score are used to evaluate patient statements and the subjective results of surgical procedures [15]. The Foot and Ankle Outcome Score (FAOS) provide valid and reliable values when assessing the prospects of revision surgery [16, 17]. However, the scores do not provide any information about the lesion’s condition. This is only possible in conjunction with new imaging. Complex statistical calculations are required for the minimum important change (MIC) if corresponding changes in the evaluation of the individual aspects are to be reliably detected [18, 19]. The MICs of the FAOS vary between different foot and ankle diagnoses and should therefore be considered in the context of therapy.

The aim of this study is to use the various scores in conjunction with imaging to identify criteria that may represent predictive factors so that patients can benefit from revision surgery. In addition, the study aims to demonstrate both postoperative symptom-free and the failure of surgical treatment.

## Materials and methods

Ethical approval for data collection and processing was granted by the local ethics committee (protocol number: 79/19), in accordance with the principles of the Declaration of Helsinki and following the STROBE guidelines for observational studies. All patients were fully informed about the study. They all gave written informed consent prior to study inclusion (Tables 1 and 2).

**Table 1:** Inhouse-Methods in percentage (%) used in relation to the OLT stage compared to ICCRA-consensus guidelines

Cartilage defect stage	Cartilage	Study (%)	Procedure
I	Intact	0%	Cartilage Substitution
		**67%**	**Bone marrow stimulation (Microfracture surgery)**
		33%	Debridement
I–II	Intact	25%	Cartilage Substitution
		**50%**	**Bone marrow stimulation (Microfracture surgery)**
		25%	Debridement
II	Intact	33%	Cartilage Substitution
	Defect	**40%**	**Bone marrow stimulation (Microfracture surgery)**
		27%	Debridement
II–III		40%	Cartilage Substitution
		**60%**	**Bone marrow stimulation (Microfracture surgery)**
III	Intact	**52%**	**Cartilage Substitution (cellular)**
		41%	Bone marrow stimulation (Microfracture surgery)
		7%	Debridement
III-IV	Defect	33%	Cartilage Substitution (cellular/acellular (AMIC® or CaReS®))
		**67%**	**Bone marrow stimulation (Microfracture surgery)**
IV	Defect	31%	Cartilage Substitution (cellular/acellular (AMIC® or CaReS®))
		**38%**	**Bone marrow stimulation (Microfracture surgery)**
		31%	Debridement

Bold, the most used technique in defect stage

**Table 2:** PROM-based surgical success by OLT stage (Patient Rating [PROMs]) in percent (%) at time of follow up

Outcome	Stage I (%)	Stage II (%)	Stage III (%)	Stage IV (%)	*p*-value
Success	33%	50%	59%	15%	0.043
Partial Improvement	25%	30%	20%	30%	0.05
No Improvement	42%	20%	21%	55%	0.008

*Abbreviations* OLT, osteochondral lesions of the talus; PROMs, patient-reported outcome measures (n = 64)

This retrospective study was conducted at the University Hospital in 2020/21 on patients treated between 2015 and 2018. In total, 64 patients underwent surgical treatment for OLT and were included and analyzed in the retrospective study. Primary outcome was a patient-level PROM-based classification (Success/ Partial relief/No relief) at follow-up. Patients were asked about their current condition, particularly regarding pain intensity, duration, location and radiation. Attention was also paid to the foot’s load-bearing capacity and limitations, including range of motion. The PROMs are based on the AOFAS. The cut-off values are those of the AOFAS score, as shown in Table 3. While delta values are missing, because preoperative PROMs were not collected. These 64 patients received 97 surgical procedures in total (Table 1). The success rate for single surgery was 67% (43/64 patients) overall, depending on the stage at time of operation (Table 2).

**Table 3:** Score classification and evaluation of surgical success based on PROM-based outcome categories in the AOFAS score

	Evaluation	AOFAS-Score (100 pts.)	AOFAS (pain) (max. 49 pts.)	FAOS (100 pts.)	VAS (10 pts.)
Excellent	Success	90–100	40 (none)	90–100	0–1
Good	Success	80–89		80–89	
Fair	Improvement	60–79	30 (less)	60–79	2–4
Poor	Failure	< 60	20 (moderate)	< 60	5–7
			10 (severe)		8–10

AOFAS, American Orthopaedic Foot & Ankle Society; FAOS, Foot & Ankle Outcome Score; VAS, visual analog scale

## Lesion characteristics

**Fig. 1:**
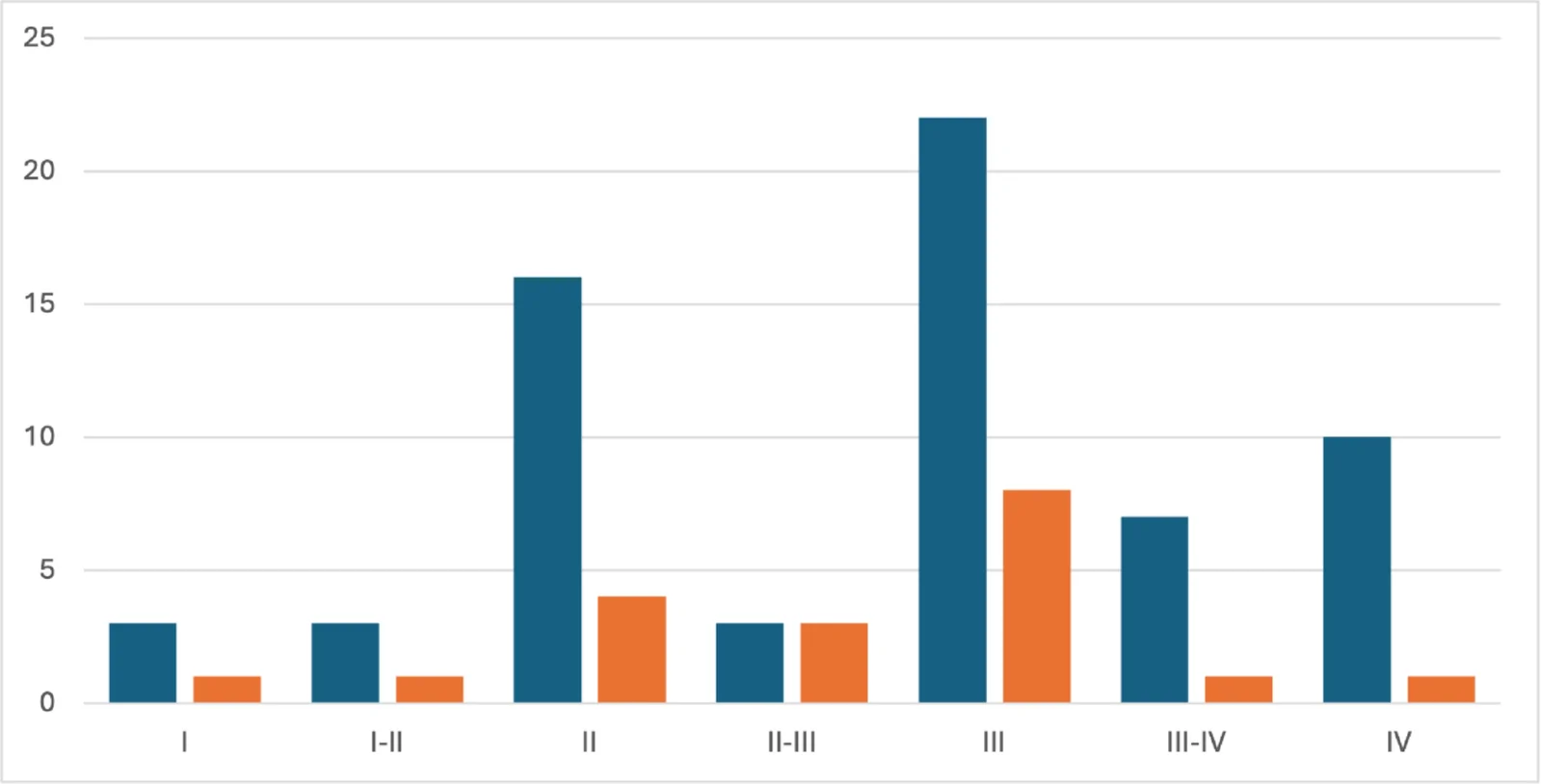
Number of patients according to OLT-grade; Column 1 (blue) = primary group, Column 2 (orange) = 21 patients of the subgroup

The average defect size for primary operations was 8.5 × 6.5 × 11.5 mm (width × depth × length), and in cases of persistent pain the defect size in revision was 8.8 × 7.2 × 12.2 mm. The defect size on the medial side was 9 × 7 × 12 mm on average; and laterally 6.5 × 5 × 10.5 mm. 37 OLTs were caused by trauma; 27 lesions had a chronic cause. The distribution by stage is shown in Fig. 1; lesions were most classified as stage 3 (together with transitional variants to stages 2 and 4).

A subgroup analysis was performed in patients with persistent symptoms and poor postoperative scores after primary OCL surgery. Inclusion criteria were persistent symptoms, pain projected to the talus, poor postoperative scores after primary surgery, and MRI evidence of an OLT in the same or worse stage. A validated scoring system such as MOCART was not used, only descriptive MRI findings. Patients with good results without further intervention were excluded. The follow-up period ranged from 2 to 5 years (mean 3 years, SD = 1.58). The mean patient age at time of surgery was 33 years (14–62 years). Five patients were treated in another hospital first. The pain in the VAS scale preoperative ranged from three to nine (mean 5.4). Eight patients report of chronic problems, 11 patients had acute injuries.

The results of the underlying study have already been discussed in detail and published under “Midterm Outcomes and Surgical Approaches for Osteochondral Lesions of the Talus” [20]. The surgical procedure was based on the stage of damage to the OCL. The operation always began with an arthroscopy of the ankle joint and, if necessary, was extended via a longitudinal incision, if more than one bone marrow stimulation / Microfracture surgery was carried out. If required, an osteotomy of the malleolus was added. No additional procedures were carried out. However, we subsequently noticed that statements about the success of surgical treatment could not be derived from this. We therefore performed a subgroup analysis. This subgroup analysis was based on patient data in the specified scores.

Patients were classified according to their clinical course at follow-up (e.g., success/partial relief/no relief; single vs. multiple surgeries). The number and type of procedures (e.g., revision with change of technique vs. revision with the same technique) were reported descriptively to characterize the course of treatment.

The success rate after a single procedure was calculated per patient. The results at follow-up were evaluated using VAS, AOFAS, and FAOS. For reasons of comparability, the AOFAS-based classification of surgical success was transferred to VAS and FAOS (Table 3).

The average age of the patients included was 33 years (range: 14–62). Inclusion criteria were persistent symptoms, pain in projection to the talus and poor postoperative scores after primary OCL surgery. Patients with good results without further intervention after treatment of OCL of the talus were excluded. In addition, an OCL in the same or worse stage had to be detectable in the MRI. The follow-up period ranged from 2 to 5 years, with a mean follow-up time of 3 years (SD = 1.58).

At the time of follow-up, outcomes were assessed using the Visual Analog Scale (VAS), AOFAS score, and FAOS. For better comparability, the classification of the AOFAS score was transferred to the VAS and FAOS (Table 3).

## Statistical analysis

Statistical analysis was performed using IBM SPSS Statistics software (Version 26.0). Due to the small group size and the lack of normal distribution, the statistical evaluation was performed using nonparametric methods after consultation with a statistician only for the surgical outcome in OLT using a chi-square test. The significance level was set at *p* ≤ 0.05 for all analyses. The group was too small to conduct a regression analysis to predict treatment factors. That means no reliable statistical conclusions can be drawn.

## Results

In 21 of 64 patients (33%), the initial operation was classified as a failure based on persistent complaints and poor postoperative scores. A new MRI scan was performed prior to the revision surgery. The surgical treatment was carried out in accordance with the radiological assessment of the lesion. Of these 21 patients, four underwent cartilage substitution (AMIC from Geistlich®, CaReS®) and in osteonecrotic defects an autologous spongiosa grafts form tibia additionally, 17 received bone marrow stimulation (included microfracture, retrograde drilling, and antegrade drilling) and two were subjected to debridement and removal of the cartilage body alone during the initial procedure. In 16 patients, the medial edge of the talus was affected; in three patients, the lateral edge was affected. An osteotomy of the medial malleolus was required in eight patients; an osteotomy of the fibula was not required in any case. These 21 patients underwent additional (multiple) surgeries, resulting in 33 revision procedures in total across the subgroup (range 2–5 procedures per patient). 25 revisions were associated with a change in procedure, and 8 revisions were performed using the same procedure. When revision strategies were summarized descriptively (change vs. same technique), the distribution of patient-rated outcomes did not indicate a statistically significant difference between strategies (Table 4). On the other hand, the groups treated with BMS showed lower success rates in percentage terms.

**Table 4:** PROM-based outcome classification at follow-up in patients with multiple procedures (n=21), according to the procedure (33 revision procedures total)

Change of procedure			
	yes	no	Total
Success	7	2	9
Improvement	7	1	8
Failed/poor	11	5	16
Total	25	8	33

At follow-up, nine of the 21 patients (43%) with multiple surgeries were symptom-free and eight patients (38%) improved at the end of the surgical treatment. They have adapted to their condition over time and manage fine in everyday life. Despite surgical treatment and multiple surgeries, four of the 21 patients (19%) did not achieve success (Table 5). These patients reported persistent or progressive symptoms and refuse further surgery.

**Table 5:** Final PROM-based outcome classification by Surgical Success based on operation strategy (single procedure vs. multiple procedures) (patient-level analysis; n = 64)

	Total success		Success single surgery		Success multiple surgery	
Success	36	56%	27	62,8%	9	43%
Improvement	16	25%	16	18,6%	8	38%
Failure/poor	12	19%	21	18,6%	4	19%
Total	64	100%	43	100%	21	100%

Firstly, patient satisfaction (surgical success) was compared with the AOFAS score, the VAS for pain and the FAOS to determine whether there was a statistical correlation between them. Subsequently, the study examined whether the two scoring systems (AOFAS score vs. FAOS score) were consistent with one another. In addition, an assessment was made to determine whether there was a difference in clinical outcome depending on the surgical strategy (revision with a change of procedure versus the same procedure). The results (in Table 4) indicate that no significant difference was found between changing the technique and retaining the same technique in terms of outcome. This was also calculated using the distribution of frequencies (chi-square test). Patient-reported success showed a significant association with the AOFAS categories (Chi^2 ^= 19.11; *p* = 0.001) and with pain on the VAS (Chi^2 ^= 52.895; *p* = 0.001). The FAOS also showed a significant correlation with patients´ subjective complaints (Chi^2 ^= 15.949; *p* = 0.003). Moreover, a significant relationship was found between the AOFAS and FAOS scores in the chi-square test (Chi^2 ^= 51.152; *p* = 0.001).

**Fig. 2:**
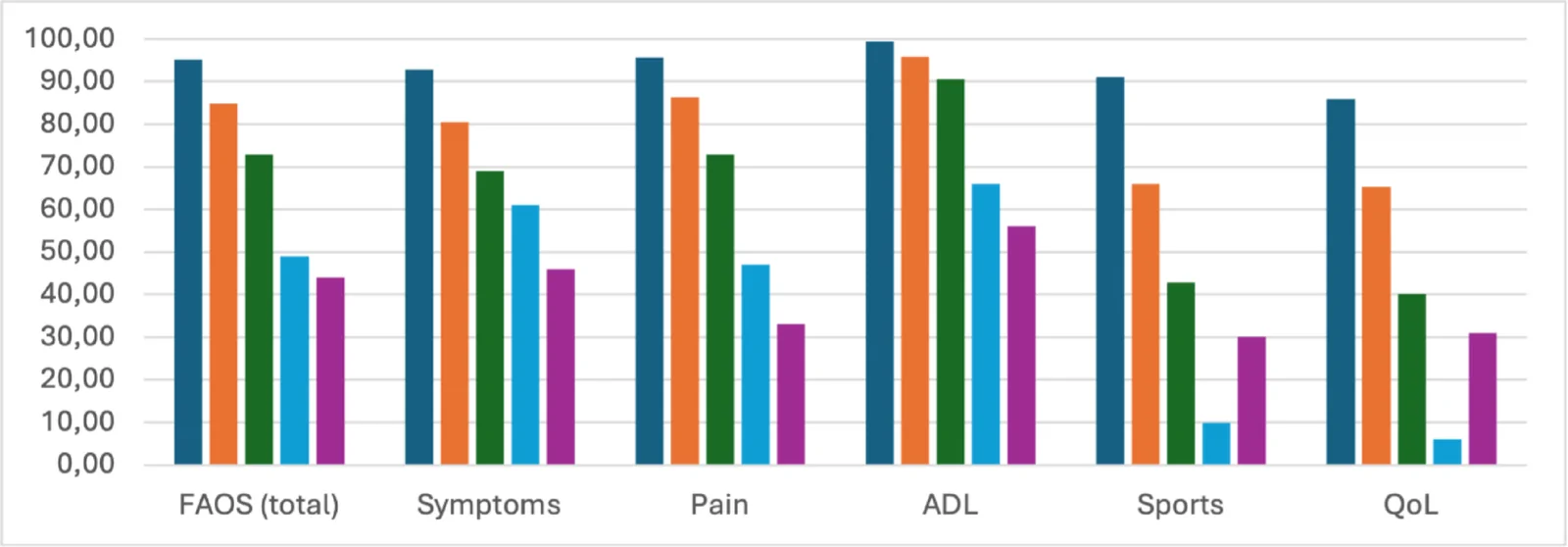
Representation of the individual subgroups (Quality of life (QoL), Sports, Activity of daily life (ADL), Pain, and Symptoms) of the FAOS score, with the individual results shown as average values (columns 1–3) in the score: Column 1 (dark blue) = excellent, Column 2 (orange) = good, Column 3 (green) = satisfactory, Columns 4 (light blue) and 5 (purple) = poor. Presenting the individual values makes the data confusing and difficult to understand and interpret the results

Although the overall scores did not serve as independent predictors of the success of revision surgery, an analysis of the FAOS subscales revealed a clear hierarchy of functional impairments. Our data suggest that the ‘Sport and Leisure’ and ‘Quality of Life’ (QoL) subscales may be the most sensitive indicators of persistent symptoms. These subscales showed declines even when the total score remained in the ‘excellent’ or ‘good’ range due to relatively stable ratings in the ‘Pain’ and ‘ADL’ (Activities of Daily Living) categories (Fig. 2, column 4 and 5). A decline in the ‘Pain’ and ‘Symptoms’ categories was only observed following a further deterioration in clinical status, whilst the ‘ADL’ scores remained stable until the ‘poor’ outcome category was reached (Fig. 2, column 4 and 5). We suspect that these sub-categories should be considered in the prognostic assessment for revision surgery to identify early signs.

**Fig. 3:**
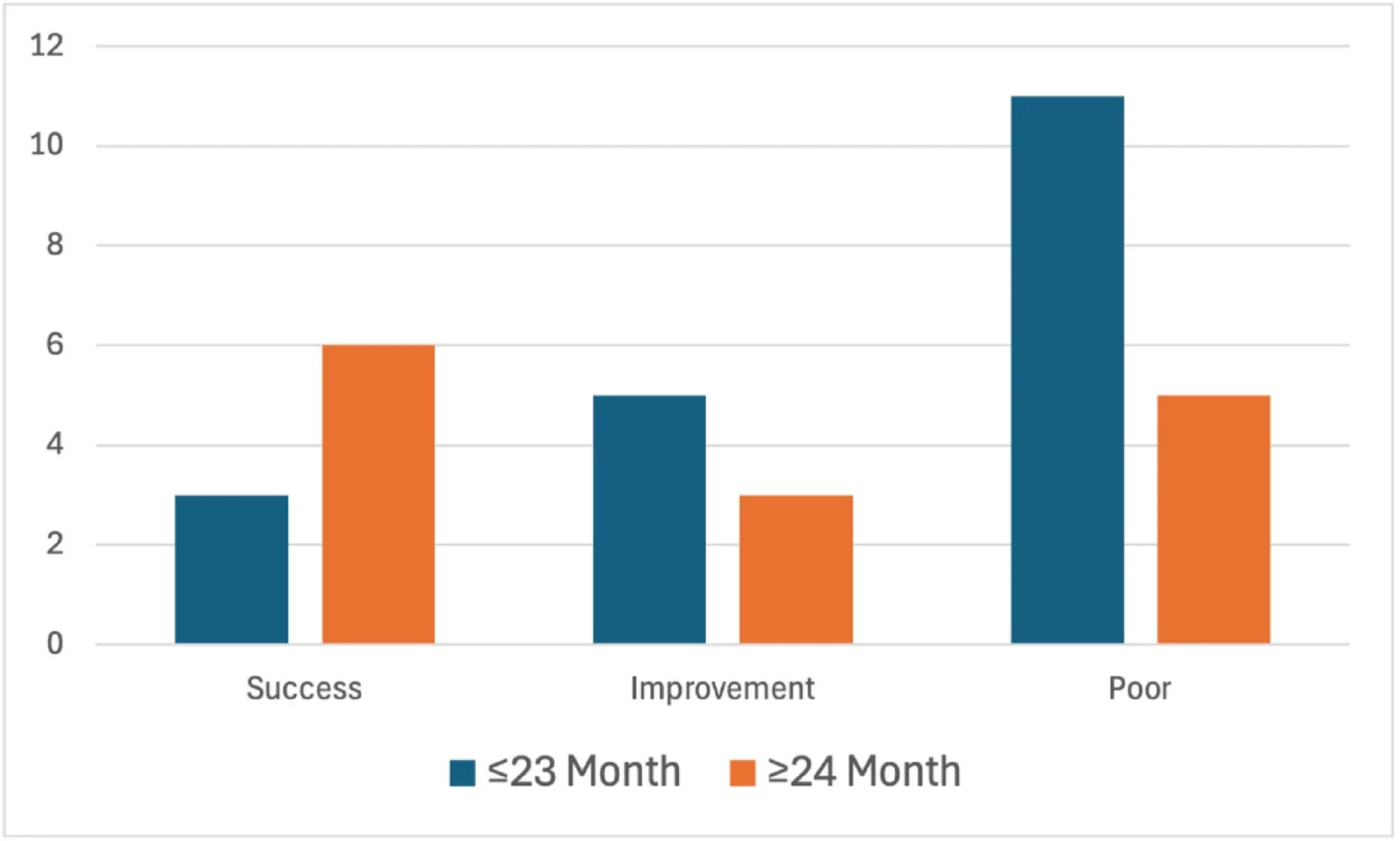
Surgical success based on postoperative waiting time until revision (n = 33)

For multiple surgeries, the average waiting time between operations was 34 months. In the cohort with a waiting time of ≥ 24 months, both the proportion of patients whose symptoms had improved and who did not require a revision, and the likelihood of a successful repeat operation in patients with persistent pain, were higher. (Fig. 3).

## Discussion

The results of this retrospective analysis of the clinical considerations for successful revision surgery for osteochondral lesions of the talus (OLT) show that a significant number of patients appear to require repeated surgical interventions and that these patients benefit from them, even though the overall success rate is moderate. The indication for revision was made based on an assessment of all available findings and the scores achieved [1]. Of the 64 patients in the original cohort, 21 continued to experience symptoms after surgery, requiring at least one further operation.

This is consistent with the data in the literature and underscores the complexity of treating OLT, which is often characterized by persistent symptoms and inadequate results after the first intervention [21, 22].

Analysis of the scores (AOFAS, FAOS, VAS) pointed out the opportunity of association between the subjective success of the patients and the objective scores. The FAOS score proved particularly valuable, as it allows for a differentiated view of the individual categories, which might be important when assessing the necessity and potential success of revision surgery [23, 24]. On the other hand revision surgery should not be based on a score in a scale nor on patient-reported pain or imaging findings alone. The relatively poor results in the early stages of the disease may be due to a bias arising from overly optimistic expectations regarding microfracture surgery. A retrospective analysis of MRI scans using the MOCART score could, in the long term, lead to an adjustment of the treatment strategy.

Interestingly, the FAOS score for these patients showed losses in specific categories such as pain and symptoms in associated with persistent problems. In contrast, losses in quality of life and sports were negligible, with the overall results here mostly excellent. This discrepancy suggests that certain aspects should be considered in cases of persistent complaints when deciding on the indication for revision. If there were losses in the QoL category, the results were poor in all scores and the prospects of success in revision surgery were low. Ramoni et al. report a significantly higher failure rate when the lesion is larger than 15 mm^2^ [25]. The instability of the subchondral bone has also been identified by various authors as a possible risk factor [26, 27]. Whilst our study focused primarily on subchondral bone grafting in combination with any necessary acellular matrix. Current trends in revision surgery are moving towards more complex joint-preserving strategies. Migliorini et al. emphasize that AMIC could be a viable option as a revision procedure in cases of failed AMIC surgery [28]. These include the use of biomimetic multilayer scaffolds for osteochondral reconstruction [29] or fresh osteochondral allografts for extensive lesions [30]. Recent studies highlight new treatment options involving augmentation with mesenchymal stromal cells or stem cells, such as filtered bone marrow aspirate (fBMA) or bone marrow aspirate concentrate (BMAC) [2, 3]. These strategies appear promising; however, they are not currently available to all those affected. On the other hand, according to Juels et al., [22] arthrodesis remains the best alternative for these patients.

The waiting time between operations also played a role in this group; a longer waiting time of more than 24 months appeared to increase the likelihood of a successful outcome in revision surgery. This could suggest that adequate postoperative rehabilitation and time for healing and adaption processes are likely to be crucial for long-term success. In addition, 19% of patients (4/21) did not experience significant improvement despite multiple surgeries. The literature considers it necessary to take preoperative factors such as age, BMI, activity level, and type of lesion into account to better predict the chances of success [31–33].

In the literature, the challenge in treating OLT is highlighted by the heterogeneity of patients and the wide range of available treatment methods [34–36]. Studies show that conservative treatments are often effective in the early stages, while the need for surgical intervention increases significantly in more advanced cases [6, 7]. Furthermore, it is emphasized that the decision to perform revision surgery should be based not only on scores, but also on clinical findings, radiological results, and the patient’s individual circumstances [13].

In conclusion, the results of this study provide possible clues into the indications for revision surgery and the treatment of OLT, highlighting the importance of a tailored assessment of the patient’s situation. Future studies should aim to investigate the long-term outcomes of revision surgery further and develop more precise guidelines for identifying suitable candidates. The use of the FAOS as an assessment tool could prove particularly helpful in better understanding the specific needs of patients and adapting treatment approaches accordingly.

## Limitations

The study has several limitations that should be considered when interpreting the results. First, its retrospective design inherently carries the risk of selection and recall bias. The lack of comprehensive preoperative baseline data for all patients limits the ability to precisely quantify the clinical improvement (delta) from the initial state to the follow-up. Second, the sample size of the revision subgroup (n=21 patients, 33 procedures) is relatively small. While this reflects clinical reality in a specialized setting, it limits the statistical power and precluded more complex analyses, such as multivariate regression, to identify independent predictors of success.

Furthermore, there is significant heterogeneity regarding the surgical techniques used in both primary and revision surgeries (ranging from microfracture to AMIC). This variety makes it difficult to isolate the efficacy of a specific surgical approach. Third, as a single-center study, the findings may reflect local surgical practices and rehabilitation protocols, which might limit the generalizability to other clinical environments. Lastly, the mean follow-up of three years represents a mid-term outcome; therefore, no definitive conclusions can be drawn regarding long-term joint preservation or the prevention of secondary osteoarthritis.

## Conclusion

In summary, our findings suggest that a multi-modal assessment—combining PROM scores (particularly the FAOS), clinical examination, and MRI findings—can provide valuable guidance when considering revision surgery for OLT. The FAOS subdomains may offer sensitive insights into specific functional deficits that justify further intervention.

Interestingly, our data indicates that a longer interval of more than 24 months between procedures may be associated with a higher likelihood of surgical success. This could potentially be attributed to completed biological remodeling processes or more rigorous patient selection over time. However, due to the exploratory nature of this study, these results should be interpreted with caution.

Future research should focus on prospective, multicenter studies with larger cohorts to validate these preliminary findings. Specifically, randomized trials comparing different revision techniques (e.g., cell-based vs. scaffold-based procedures) and the impact of standardized postoperative rehabilitation protocols are needed. Long-term studies would allow for evaluating the efficacy of revision pathways in preventing end-stage ankle osteoarthritis.

## Data Availability

The data sets used and/or analyzed during the current study are available from the corresponding author on reasonable request.

## Abbreviations

ADL: Activity of daily life
AMIC: Autologous matrix-induced chondrogenesis
AOFAS: American Orthopedic Foot and Ankle Society
BMI: Body mass index
FAOS: Foot and ankle outcome score
ICCRA: International consensus meeting on cartilage repair of the ankle
MIC: Minimum important change
OCL: Osteochondral lesions
OLT: Osteochondral lesions of the talus
PROMs: Patient-reported outcome measures
QoL: Quality of life
VAS: Visual analog scale
